# Molecular Testing Identifies Determinants of Exceptional Response and Guides Precision Therapy in a Patient with Lethal, Treatment-emergent Neuroendocrine Prostate Cancer

**DOI:** 10.7759/cureus.5197

**Published:** 2019-07-22

**Authors:** Claire B Turina, Daniel J Coleman, George V Thomas, Alice W Fung, Joshi J Alumkal

**Affiliations:** 1 Oncology, Knight Cancer Institute, Oregon Health & Science University, Portland, USA; 2 Pathology, Knight Cancer Institute, Oregon Health & Science University, Portland, USA; 3 Diagnostic Radiology, Oregon Health & Science University, Portland, USA

**Keywords:** treatment-emergent neuroendocrine prostate cancer, homologous recombination, precision medicine, molecular testing, breast cancer type 2 susceptibility protein (brca2)

## Abstract

Nearly all prostate cancers start out as adenocarcinomas driven by the androgen receptor (AR). Neuroendocrine prostate cancer (NEPC) is a rare, AR-independent subtype with a poor prognosis and limited treatment options. Importantly, because of the widespread use of novel AR-targeting agents, the incidence of treatment-emergent (t)-NEPC is increasing in frequency. Molecular features commonly found in prostate adenocarcinomas are now well-recognized, including defects in homologous recombination (HR) genes, like breast cancer type 2 susceptibility protein (BRCA2), leading to increased sensitivity to deoxyribonucleic acid (DNA)-damaging agents (e.g., platinum chemotherapy or poly adenosine diphosphate-ribose polymerase (PARP) inhibitors). However, our own prior work demonstrates that HR gene defects are uncommon in t-NEPC. Herein, we describe a patient who originally presented with adenocarcinoma but who subsequently developed t-NEPC. Molecular testing determined that his t-NEPC tumor (but not his original adenocarcinoma) harbored complete copy number loss of BRCA2, as well as copy number loss of another HR gene - ataxia telangiectasia, mutated (ATM). Uncharacteristically for t-NEPC, the patient achieved a complete response to platinum chemotherapy. Based on emerging data for the role of maintenance PARP inhibitor therapy in ovarian cancer patients whose tumors harbor BRCA1/2 defects, we treated him with PARP inhibitor maintenance after chemotherapy. At nine months follow-up, the patient was still in complete remission. This report demonstrates the importance of molecular testing to clarify the biology of exceptional responders and to direct treatment. Our results also suggest that clinical trials of PARP inhibitor maintenance may be warranted in select patients with advanced prostate cancer, including those with t-NEPC, whose tumors harbor HR defects.

## Introduction

Neuroendocrine prostate cancer (NEPC) is a rare, androgen receptor (AR)-independent prostate cancer subtype with a poor prognosis and limited treatment options. While diagnoses of de novo NEPC are extremely rare, the incidence of treatment-emergent NEPC (t-NEPC) has risen with the widespread use of potent AR-targeted agents, such as enzalutamide and abiraterone, in men with metastatic castration-resistant prostate cancer (CRPC) [1]. Indeed, the West Coast Prostate Cancer Dream Team recently determined that 17% of metastatic CRPC biopsies harbor this aggressive t-NEPC phenotype. Prior analysis identified molecular features present in subsets of NEPC, including loss of function of the retinoblastoma (RB1) and/or tumor protein 53 (TP53) tumor suppressor genes; however, defects in genes of the homologous recombination (HR) pathway, such as breast cancer type 2 susceptibility protein (BRCA2), were rarely seen [1]. This differs from the well-recognized molecular profile of CRPC adenocarcinomas in which defects in HR genes are more commonly found and may lead to increased sensitivity to deoxyribonucleic acid (DNA)-damaging agents (e.g., platinum chemotherapy or poly adenosine diphosphate-ribose polymerase (PARP) inhibitors) [2].

Whether presenting de novo or emerging after androgen deprivation therapy (ADT), NEPC has poor survival and limited treatment options [3]. Commonly, patients with NEPC are treated with regimens used in small cell lung cancer platinum-based chemotherapy. However, the duration of benefit with chemotherapy is short-lived, and there are no other known effective treatment options for NEPC patients, underscoring the urgent need to identify treatments that can augment the anti-tumor activity of chemotherapy.

Herein, we report a 75-year-old man who developed t-NEPC after an original diagnosis of prostate adenocarcinoma. Importantly, molecular testing determined that his t-NEPC tumor (but not his original adenocarcinoma tumor) harbored complete BRCA2 copy number loss and ataxia-telangiectasia mutated (ATM) copy number loss. He achieved an exceptional and durable response to platinum-based chemotherapy and was treated with PARP inhibitor maintenance following chemotherapy, leading to persistent remission. These results demonstrate the value of molecular testing to understand the mechanisms of exceptional response and to direct precision therapy in patients with metastatic CRPC, including those with t-NEPC. 

## Case presentation

Institutional review board (IRB) approval (IRB #11025) was obtained prior to performing a review of this patient’s medical record. The subject provided written, informed consent for this chart review and molecular testing.

Initial presentation

In November of 2015, the patient presented with an elevated prostate-specific antigen (PSA) of 9.23 ng/mL but a normal prostate exam (Stage cT1c). A prostate biopsy revealed prostate adenocarcinoma involving five of 12 cores with a Gleason score of 3 + 3 = 6. Three months later, he underwent a radical prostatectomy. His pathology showed bilateral prostate adenocarcinoma with a Gleason score of 4 + 4 = 8. He had bilateral involvement of the seminal vesicles, but all of the nine lymph nodes removed were free of cancer (Stage pT3bN0).

Initial diagnosis and treatment of prostatic adenocarcinoma

The patient's postoperative PSA after surgery only declined to 1.38 ng/mL. Though salvage radiation therapy was considered, the decision was made to treat with a gonadotropin-releasing hormone (GnRH) agonist alone due to the high postoperative PSA level. He remained on treatment for one year but never experienced a PSA decline. The persistent PSA rise in the setting of a castrate testosterone was consistent with a diagnosis of CRPC that was primary and refractory to GnRH agonist therapy. The patient underwent restaging, and a computerized tomography (CT) scan showed new external iliac and pelvic sidewall lymphadenopathy, as well as an L3 sclerotic lesion that was confirmed on a technetium bone scan. The AR antagonist, enzalutamide, was added to his GnRH agonist. After one month of enzalutamide treatment, the patient’s PSA fell from 17.8 ng/mL to 0.2 ng/mL. However, the response was short-lived, and his PSA steadily rose to 3.4 ng/mL two months later.

**Figure 1:**
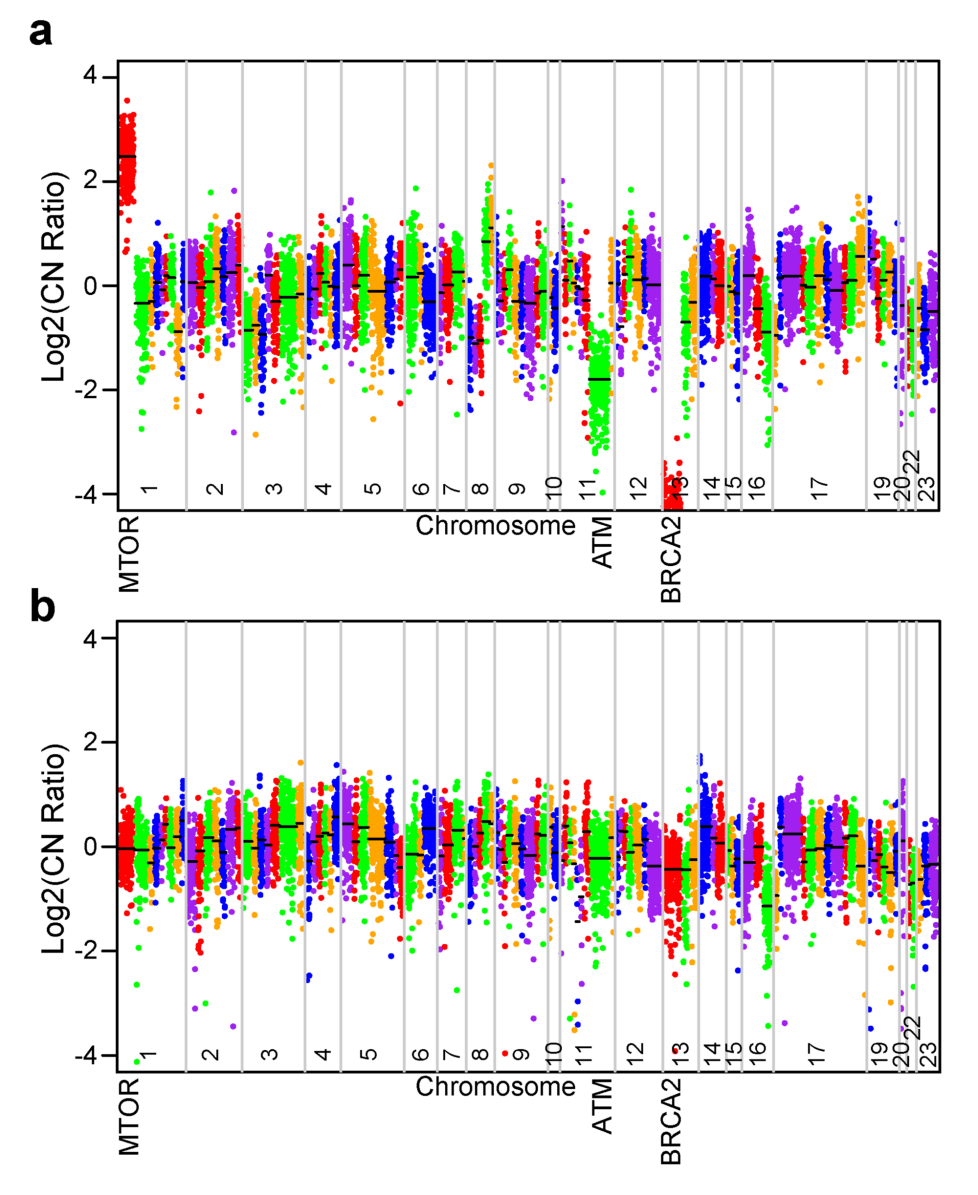
Genomic abnormalities identified in the t-NEPC sample compared to the baseline adenocarcinoma sample A) Copy number plot of the t-NEPC sample taken from the metastatic site at the neck of the bladder; B) Copy number plot of patient’s adenocarcinoma sample from his radical prostatectomy for comparison ATM: ataxia telangiectasia mutated; BRCA2: breast cancer type 2 susceptibility protein; CN: copy number; MTOR: mammalian target of rapamycin; t-NEPC: treatment-emergent neuroendocrine prostate cancer

Four months after starting enzalutamide, his PSA rose to 9.93 ng/mL, and restaging scans showed new and enlarging lymphadenopathy, hepatic lesions, and a mass extending into his bladder neck that caused pelvic pain and hydronephrosis of the right kidney. A cystoscopically-guided biopsy of the bladder mass showed small cell histology. Immunohistochemical analysis demonstrated tumor cells that were positive for synaptophysin and chromogranin and the Ki-67 was 50%, all of which were consistent with a diagnosis of t-NEPC. The patient’s t-NEPC tumor was sent for molecular testing using the Knight Diagnostic Lab GeneTrails® (Knight Diagnostic Lab, Portland, OR) comprehensive solid tumor panel of 124 genes commonly altered in cancer. Testing demonstrated a complete copy number loss of the BRCA2 locus, ATM copy number loss, Rb1 copy number loss, and mammalian target of rapamycin (mTOR) gene amplification (Figure 1a). To determine if these genetic alterations were present at diagnosis, we sequenced his original adenocarcinoma primary tumor from his radical prostatectomy sample. The radical prostatectomy specimen had 70% tumor but revealed no alterations in any of the genes examined, including BRCA2, ATM, Rb, or mTOR (Figure 1b).

Treatment-emergent neuroendocrine prostate cancer management

**Figure 2:**
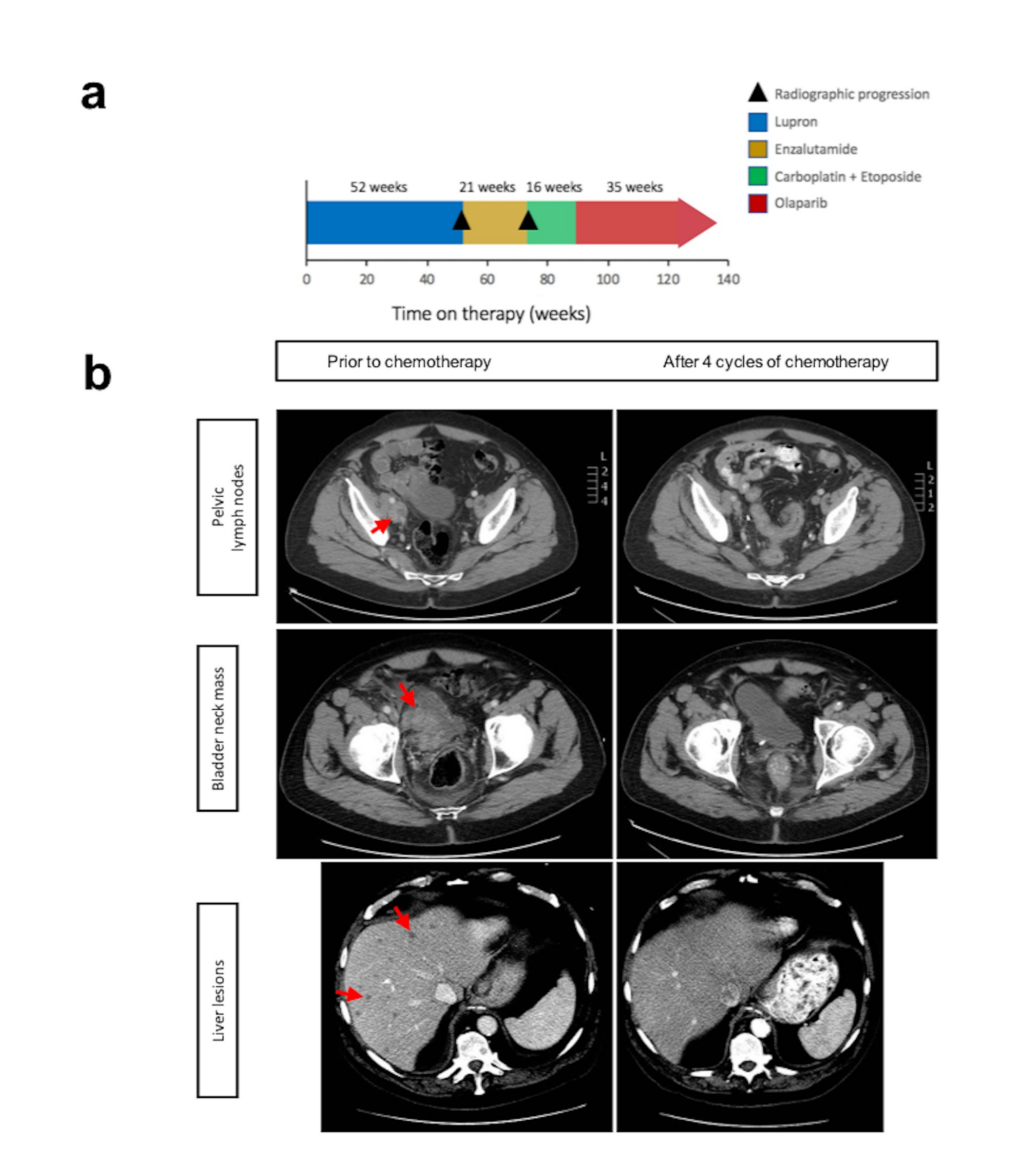
Overview of this patient’s treatment history and radiographic response to platinum chemotherapy A) Swimmer’s plot summarizing the patient’s treatment course from diagnosis through PARP inhibitor treatment, including time on treatment and reason for treatment discontinuation; B) Computed tomography images after four cycles depicting the patient’s radiographic response to platinum-based chemotherapy. Metastatic lesions in the pre-treatment scan are indicated by an arrow. PARP: poly adenosine diphosphate-ribose polymerase

After the diagnosis of t-NEPC, the patient was started on combination chemotherapy with carboplatin area under curve (AUC) 5 and etoposide, 100 mg/m^2^ every three weeks (Figure 2a). After two cycles, his pelvic pain resolved, and a CT scan showed significant reduction of the retroperitoneal lymphadenopathy and hepatic lesions, along with resolution of the right-sided hydronephrosis (Figure 2b). After his fourth cycle of chemotherapy, his PSA was undetectable (< 0.1 ng/mL), and imaging studies showed complete resolution of the hepatic lesions and lymphadenopathy with no new metastatic sites (Figure 2b). The patient received two more cycles of chemotherapy for a total of six cycles. After treatment completion, his technetium bone scan showed stability of his L3 lesion. His CT scans were without evidence of metastatic disease, and his PSA remained undetectable, consistent with a durable remission.

Because of the BRCA2-null status of his tumor, the high risk for relapse after chemotherapy alone, and because of emerging literature in ovarian cancer demonstrating the benefit of PARP inhibitor maintenance in those with BRCA1 or BRCA2-deficient tumors after platinum-based chemotherapy, we elected to start treatment with the PARP-inhibitor, olaparib (300 mg twice per day), as maintenance therapy. After two months, he was dose-reduced to 150 mg twice per day due to intolerable toxicities of fatigue and nausea. After nine months of olaparib, his PSA remained undetectable, and his technetium bone scan and CT scans were also without evidence of recurrence.

## Discussion

t-NEPC is an aggressive prostate cancer subtype for which effective treatments are lacking. Until recently, the incidence of t-NEPC was unknown, but we now know that approximately 17% of patients with CRPC who underwent metastatic biopsies had tumors harboring t-NEPC [1]. The mechanisms responsible for the switch from adenocarcinoma to t-NEPC are poorly understood, and it is still unclear whether t-NEPC occurs because of the selection of a preexisting clone vs. transdifferentiation of adenocarcinoma cells that acquire new genetic or epigenetic alterations with ADT, such as those seen in this patient.

Unlike adenocarcinoma CRPC tumors, defects in HR DNA repair genes, such as BRCA2, were felt to be rare in t-NEPC [1, 4]. HR DNA repair defects are important because cells deficient in both copies of an HR DNA repair gene are incapable of repairing DNA damage using non-error-prone mechanisms [5]. Rather, cells harboring dual defects in an HR DNA repair gene are forced to use the error-prone, non-homologous end joining (NHEJ) pathway to repair DNA strand breaks. This error-prone pathway can result in additional deleterious mutations, culminating in apoptosis. For this reason, cancer cells harboring complete loss of HR DNA repair genes are particularly susceptible to treatment with DNA damaging chemotherapy, such as platinum salts, that introduce double-strand breaks [6].

Recently, the use of PARP inhibitors to treat cancer has been gaining interest. The PARP1 protein is required for repair of DNA strand breaks and damage at the replication fork [7]. Inhibition of PARP proteins by themselves does not result in cell death; however, when PARP proteins are inhibited in cells with loss of HR DNA repair genes, this can lead to cell death - a concept known as synthetic lethality [8-9]. Importantly, PARP inhibitors have already been shown to improve progression-free survival in women with ovarian cancer, especially in those with BRCA1/2 alterations, when used as maintenance after platinum chemotherapy and in women with breast cancer with germline BRCA1/2 alterations who have received multiple lines of prior chemotherapy [10-15]. Moreover, recent trials in metastatic CRPC also demonstrate that PARP inhibitors may improve outcomes for metastatic CRPC patients, especially in those with dual defects in BRCA2 [16-18]. These results prompted us to consider maintenance olaparib in the patient described herein, which may have contributed to his persistent remission.

Importantly, this report provides evidence that HR DNA repair defects may be detected in t-NEPC patient tumors. The complete loss of BRCA2 in this patient’s tumor likely contributed to his exceptional response to platinum-based chemotherapy and continued remission while on PARP inhibitor maintenance. Similar durable responses to platinum chemotherapy and olaparib have also been reported in other patients with BRCA2-null NEPC [19-20]. Our patient’s tumor also harbored ATM copy number loss, though one allele appeared to still be intact. We cannot be certain if the remaining ATM allele was still expressed and functional or if epigenetic mechanisms also contributed to the loss of the remaining ATM allele. Thus, while it is uncertain whether this patient’s ATM gene alteration contributed to his response, dual defects in an HR gene are felt to be necessary for platinum or PARP inhibitor response [8, 16-17].

Another limitation of our study is that we do not know when this patient’s tumor became HR-deficient. Single-cell sequencing of his original tumor could have helped to address this question, but this was not possible because the patient’s original tumor was a paraffin-embedded, archived sample. Thus, it is unclear whether the BRCA2, ATM, and mTOR mutations we identified in his t-NEPC tumor were preexistent in rare clones or acquired through resistance to ADT or enzalutamide.

Currently, there are several clinical trials investigating PARP inhibitors for the treatment of CRPC in patients whose tumors harbor mutations in HR DNA repair genes. However, there are no clinical trials investigating PARP inhibitors specifically in t-NEPC patients, and t-NEPC patients are often excluded from CRPC clinical trials due to concerns about rapid progression.

## Conclusions

This case report demonstrates that HR DNA repair alterations may be found in t-NEPC patient tumors and demonstrates the importance of molecular testing to clarify the biology of exceptional responders and to direct precision oncology treatment. Our results also suggest that clinical trials of PARP inhibitor treatment or PARP inhibitor maintenance after platinum chemotherapy may be warranted in select patients with advanced prostate cancer, including those with t-NEPC who achieve exceptional responses to platinum chemotherapy whose tumors harbor loss of function alterations in HR DNA repair pathway genes.
